# An alternative real-time fluorescence reverse transcription loop-mediated isothermal amplification assay for the rapid detection of SARS-CoV-2

**DOI:** 10.1590/S1678-9946202567037

**Published:** 2025-06-27

**Authors:** Janisara Rudeeaneksin, Wiphat Klayut, Benjawan Phetsuksiri, Ballang Uppapong, Thanee Wongchai, Nuttagarn Chuenchom

**Affiliations:** 1 Ministry of Public Health, National Institute of Health, Department of Medical Sciences, Nonthaburi, Thailand; 2 Ministry of Public Health, Medical Sciences Technical Office, Department of Medical Sciences, Nonthaburi, Thailand; 3 Ministry of Public Health, Administrative Office, Department of Medical Sciences, Nonthaburi, Thailand; 4 Ministry of Public Health, Mae Sot Hospital, Tak, Thailand

**Keywords:** COVID-19, SARS-CoV-2, RT-LAMP, RT-PCR, Fluorescence

## Abstract

COVID-19, caused by SARS-CoV-2 virus infection, remains a public health concern in many countries. Reverse transcription loop-mediated isothermal amplification (RT-LAMP) is a rapid and cost-effective alternative test for COVID-19 diagnosis. In this study, we developed and evaluated a real-time RT-LAMP (rRT-LAMP) assay coupled with a melting curve analysis to detect SARS-CoV-2. The reaction was carried out in a real-time thermal cycler at 63 °C for 45 min to amplify the region of SARS-CoV-2 *orf8*; real-time monitoring of amplification was performed by fluorescence detection. The performance was assessed by comparing it to a real-time reverse transcription-polymerase reaction (rRT-PCR) as a reference. The rRT-LAMP could detect as few as 15 copies of SARS-CoV-2 RNA per reaction. Positive results appeared within 30 min, while the melting-temperature analysis could verify the amplification specificity. No positive results from non-SARS-CoV-2 templates and no mis-amplification were observed. The comparative analysis using 262 RNA extracted from nasopharyngeal swab samples revealed the overall accuracy, sensitivity, specificity, positive predictive value (PPV), and negative predictive values (NPV) of the rRT-LAMP at 88.55% (95% CI: 77.52–100%), 84.13% (95% CI: 71.56–98.27%), 100% (95% CI: 78.38–100%), 100% (95% CI: 85.06–100%), and 70.87% (95% CI: 55.55–89.11%), respectively. The greatest sensitivity was as high as 98–100% for specimens with threshold rRT-PCR cycle (Ct) values of less than 30 cycles. Overall, this rRT-LAMP showed good performance for the rapid detection of SARS-CoV-2. It is proposed as a potential method for real-time amplification detection, offering increased laboratory capacity for SARS-CoV-2 testing in a cost-effective and timely manner.

## INTRODUCTION

The coronavirus disease 2019, or COVID-19, an infectious disease caused by the severe acute respiratory syndrome coronavirus-2 (SARS-CoV-2), has resulted in millions of infections globally since its first outbreak in December 2019^1^. In May 2023, the World Health Organization (WHO) declared the end of the global COVID-19 pandemic^2^. During the declining phase of COVID-19, less concern for early case identification and individual prompt separation from suspected infections combined with inadequate immunity has led to a resurgence in COVID-19 and the spread of infection. Although COVID-19 cases have reduced, the disease continues to pose a public health issue to communities in many countries, including Thailand, impacting global health and the economy. For effective patient care and COVID-19 control, diagnostic testing—including rapid case identification and notification of infection—remains crucial.

Real-time reverse transcription-polymerase chain reaction (rRT-PCR) is considered the gold standard for COVID-19 diagnosis due to its speed and high accuracy^3,4^. However, rRT-PCR testing is costly, time-consuming, and relatively complicated, requiring trained laboratory professionals to perform it. These issues limit its use to only well-equipped laboratories with access to real-time thermal cyclers^5,6^. Meanwhile, various methods have been developed to rapidly detect SARS-CoV-2 infection^7,8^. Nowadays, SARS-CoV-2 antigen rapid tests and other simpler diagnostics for simple, fast, and cheap COVID-19 detection have become readily available and are often used as point-of-care or for self-testing at home^9^. The commonly used antigen rapid tests are inexpensive, easy to perform, and yield results within 15–30 min. Nevertheless, the major limitation is their lower sensitivity compared to rRT-PCR^10^. The detection limit for viral copies is reported to be at least 2–3 times greater than rRT-PCR^11^. In this instance, other rapid and cost-effective molecular assays could be alternatively used for the efficient diagnosis of COVID-19.

Loop-mediated isothermal amplification (LAMP) represents a simple and rapid molecular test that reliably detects target sequences by nucleic acid amplification through isothermal conditions^12,13^. The sensitivity and specificity of the LAMP technique are comparable to PCR, while the total turnaround time until the results come out is usually less than an hour^14^. Unlike PCR, the rRT-LAMP reaction uses strand displacement polymerase and a set of four to six primers to amplify a particular DNA sequence in a single step at a constant temperature ranging 60-65 °C within a short time^15,16^. Reverse transcription-LAMP (RT-LAMP) combines reverse transcription and LAMP reactions, which co-occur to detect viral RNA^15^.

The RT-LAMP techniques are versatile and highly adaptable while retaining an acceptable level of sensitivity and specificity for faster detection of specific sequences compared to RT-PCR. The amplification results can be identified by various means, such as direct visual observation of the color change, turbidity detection, fluorescence measurements, or different combined methodologies^17,18^. The colorimetric RT-LAMP assays enable a simple readout of results via color change visualization, providing an easy and inexpensive method for use in various settings, including laboratories with limited resources^19,20^. To date, numerous RT-LAMP-based assays have been developed to detect SARS-CoV-2. Most assays found in the literature use colorimetric RT-LAMP assays with varied sensitivity for the detection of SARS-CoV-2 RNA. Previously, we reported a visual RT-LAMP based on pH change, enabling simple and low-cost applications with good sensitivity and specificity for the rapid detection of SARS-CoV-2^21,22^. Since rRT-PCR is a standard reference for COVID-19 diagnosis, real-time thermal cyclers are now more accessible in many laboratories. These real-time thermocyclers can be used to carry out fluorescent rRT-LAMP assays, enabling result monitoring in a similar real-time manner to rRT-PCR, but at a lower cost and shorter duration. Considering these advantages, we sought to develop and validate a simple rRT-LAMP assay coupled with melting curve analysis for real-time monitoring of the amplification to detect SARS-CoV-2. The clinical performance was assessed by comparing the detection results against rRT-PCR as a reference.

## MATERIALS AND METHODS

### Study setting and design

This study was carried out at the National Institute of Health (NIH), the Department of Medical Sciences, and Mae Sot Hospital, a community hospital with 365 inpatient beds in the Tak province, close to the Thailand-Myanmar border. The rRT-LAMP assay was developed, and nasopharyngeal swab (NPS) specimens were used in clinical evaluation. All procedures performed with the clinical samples in this study were conducted following ethical guidelines and regulations. The study protocol was reviewed and approved by the Ethics Committee of Mae Sot Hospital, Ministry of Public Health, Thailand (Reference MSHP 024/2564).

### rRT-LAMP assay

#### Oligonucleotide rRT-LAMP primers

The oligonucleotide rRT-LAMP primers targeting SARS-CoV-2 *orf8* reported in our previous development were used in this study^21^. A primer set consisted of an outer forward primer (F3), an outer backward primer (B3), a forward inner primer (FIP), a backward inner primer (BIP), a loop forward primer (LF), and a loop backward primer (LB). Before testing, a 10X primer mix consisting of 16 μM FIP and BIP, 2 μM F3 and B3, and 4 μM LF and LB in nuclease-free water was prepared, and stored at −20 °C until use.

#### rRT-LAMP reaction

A simple rRT-LAMP assay was set up using readily available LAMP reagents purchased from New England Biolabs (NEB, Ipswich, MA, USA). Each reaction comprised a volume of 20 µL of the pre-mix consisting of 12.5 µL of WarmStart^®^ 2X LAMP Master Mix, 4.5 μL of nuclease-free water, 0.5 μL of 50X fluorescence dye, and 2.5 μL of 10X primer mix, with each 5 μL of the RNA extract added at the final step, immediately before starting the reaction. The reaction mixture was incubated at 63 °C for 45 min in a real-time PCR analyzer (CFX96™ Real-Time PCR Detection System, Bio-rad, Hercules, CA, USA). The real-time amplification detection was set up for 45 cycles with signal acquisition every minute, and the FAM channel (450–490 nm for excitation, 510–530 nm for detection) was used to read the reporter dye. Amplification signals were measured using relative fluorescence units (RFU) per cycle with the same real-time PCR instrument; all rRT-LAMP data acquired were analyzed by the Bio-Rad CFX96™ Software. The negative control contained nuclease-free water without a SARS-CoV-2 RNA template. For validated results, the positive control reactions show the specific amplification curve, and the negative control reactions have no amplification curve. The amplified products were further verified using the melting curve analysis.

### Melting curve analysis of rRT-LAMP amplicons

Melting curves or dissociation curves were analyzed after the completion of amplification to characterize and verify the rRT-LAMP amplicons. To perform the melting curve analysis, the real-time PCR detection system was programmed to include a melting profile immediately following the isothermal amplification protocol. At post-amplification, the real-time thermal cycler reheated the amplified products to generate melting temperature data. In the study protocol, the dissociation temperature range was set up to extend from 63 °C to 95 °C followed by temperature decrement. The rRT-LAMP detection was considered SARS-CoV-2 positive if a characteristic peak on the melting curve of the amplification product specific to the target sequence was observed. Otherwise, the sample was considered negative for SARS-CoV-2 RNA.

### Determination of analytical sensitivity and specificity

The analytical sensitivity of the rRT-LAMP was determined using AccuPlex™ SARS-CoV-2 Reference Material Kit (SeraCare, Milford, MA, USA) as a reference material for SARS-CoV-2 RNA. Due to the limited amount of reference RNA, the serial dilutions of the RNA material were prepared in the reaction mixture with the target concentrations of SARS-CoV-2 RNA templates at 5,203 (original concentration), 4,000, and 3,000 copies/mL. Each dilution was subjected to rRT-LAMP assay at 63 °C for 45 min. The rRT-LAMP amplification curves were detected, and the melting curve (dissociation curve) of the amplified products was analyzed. The SARS-CoV-2 BA.2.75 variant, a known genotype, was also included as a reference virus isolate to detect different SARS-CoV-2 strains.

As described previously, the specificity of primers was assessed *in silico* by analyzing the homology of primers used with the genomes of closely related human, viral and bacterial pathogens causing respiratory infections^21^. Moreover, RNA samples from related coronaviruses and other common respiratory viruses—including Influenza A, H1, and B (Flu A H1, Flu B), respiratory syncytial virus A and B (RSV A, B), and Middle East respiratory syndrome (MERS)—were tested by the rRT-LAMP, and SARS-CoV-2 RNA was used as the positive control.

### Sample collection and handling

RNA materials from NPS specimens were used to evaluate the clinical performance of the rRT-LAMP. The NPS specimens were taken from suspected cases of COVID-19 and kept in a 2-mL viral transport medium (VTM). The collected NPS samples in VTM-containing tubes were transported in safety containers at 4 °C, delivered to the laboratory, and tested by regular rRT-PCR in the diagnostic laboratory of Mae Sot Hospital.

The NPS residual samples for the rRT-LAMP analysis were anonymized without patients’ information, namely: name, age, gender, and clinical-pathological characteristics.

### Detection of SARS-CoV-2 by rRT-PCR

The rRT-PCR testing was performed as part of the routine diagnostic practice for COVID-19 patients’ care at Mae Sot Hospital. Briefly, the total RNA was extracted from 200 µL of NPS-suspended VTM using Zybio^®^ nucleic acid extractor with the Zybio^®^ nucleic acid extraction kit (Zybio Inc., Chongqing, China). The RNA materials were finally eluted in 200 µL of elution buffer provided with the kit. The Molaccu^®^ COVID-19 RT-PCR Detection Kit (Zybio Inc., China) was used to detect SARS-CoV-2 RNA following the manufacturer's instructions. This assay amplifies portions of RNA-dependent RNA polymerase (*RdRp*), nucleocapsid (*N*), (SARS-CoV-2), and envelope *(E*) genes (Sarbecovirus) specific to SARS-CoV-2. The rRT-PCR was carried out in the CFX96™ real-time PCR detection system. Samples were considered positive for SARS-CoV-2 when the amplification cycle threshold (Ct) value was ≤ 40, at least two targets were detected, and the amplification curves exhibited a typical S-shape.

After rRT-PCR testing, the NPS residual samples were carefully stored and transported in a box with dry ice to the NIH laboratory in Thailand for subsequent rRT-LAMP analysis.

### Detection of SARS-CoV-2 by rRT-LAMP

For rRT-LAMP testing, viral RNA was extracted from 200 µL of stored NPS residues in VTM using NucleoSpin RNA Virus (Macherey-Nagel GmbH & Co. KG, Germany) according to the manufacturer's instructions. The RNA sample was finally eluted in 30 µL of elution buffer provided with the kit. The resulting RNA was used as RNA templates for detecting SARS-CoV-2 by the rRT-LAMP assay and stored at −80 °C until use. The rRT-LAMP reaction was carried out as previously described.

### Data and statistical analysis

The data were presented in the form of numbers or percentages with 95% confidence intervals (CIs). Diagnostic test performance was summarized as accuracy, sensitivity, specificity, positive predictive value (PPV), and negative predictive value (NPV). which were determined by comparing the rRT-LAMP results to those of the reference rRT-PCR test, and then calculated using Medcalc (version 23.2.1, MedCalc Software Ltd., Ostend, Belgium).

## RESULTS

### rRT-LAMP assay for real-time detection of SARS-CoV-2 RNA

**Figure 1:**
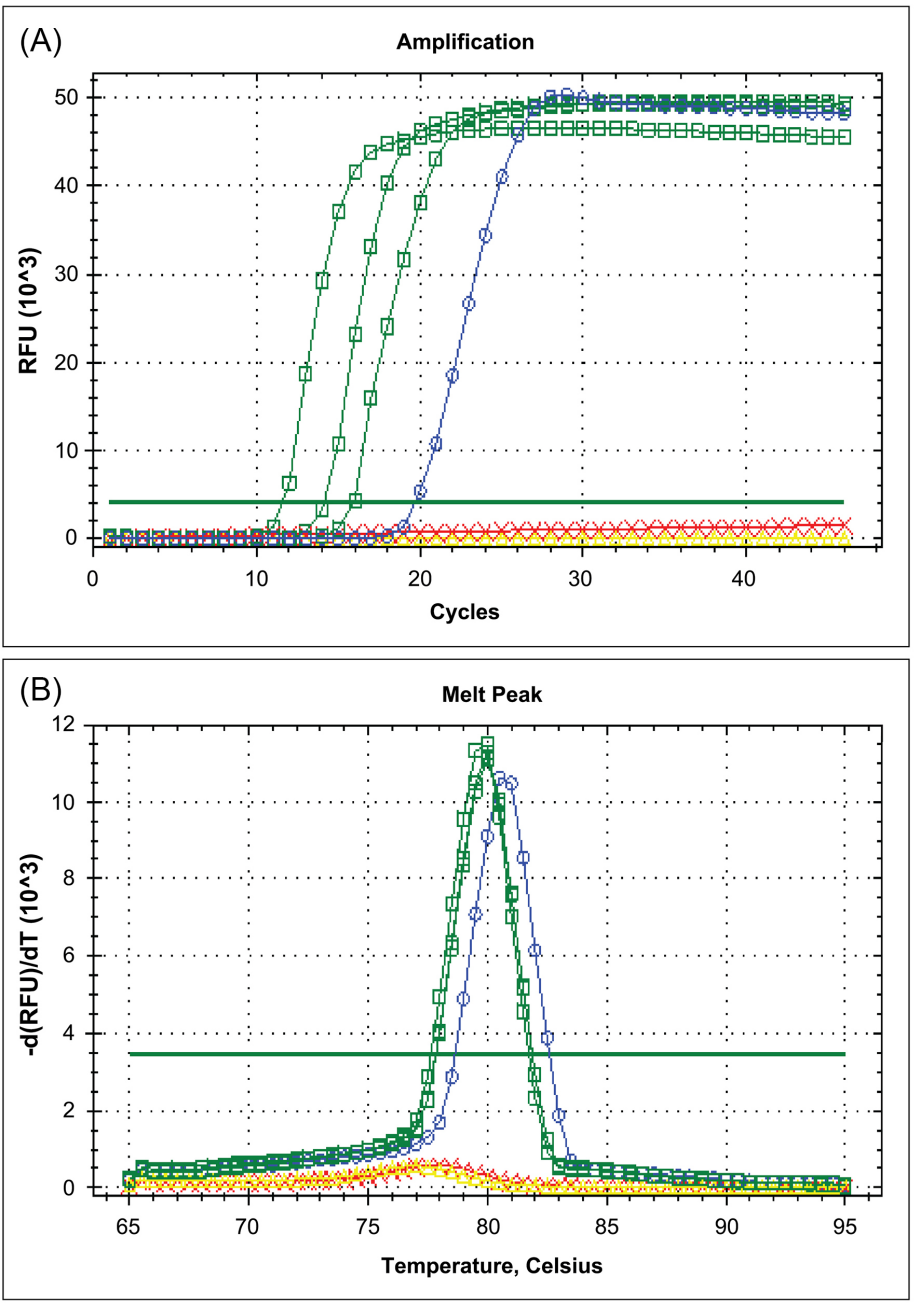
The examples of rRT-LAMP results for the detection of SARS-CoV-2. A) Amplification curves of fluorescence-based rRT-LAMP targeting SARS-CoV-2 *orf8.* B) Results of the melting curve analysis showing the melting peaks detected in the range of 79.5–80.5 ± 1 °C. (Cycle number on the x-axis corresponded to the reaction time in minutes, and the detection setup was 45 cycles of 1 minute each. Blue curve line: positive control; red curve line: negative control; green curve lines: SARS-CoV-2 positive samples).

**Figure 2:**
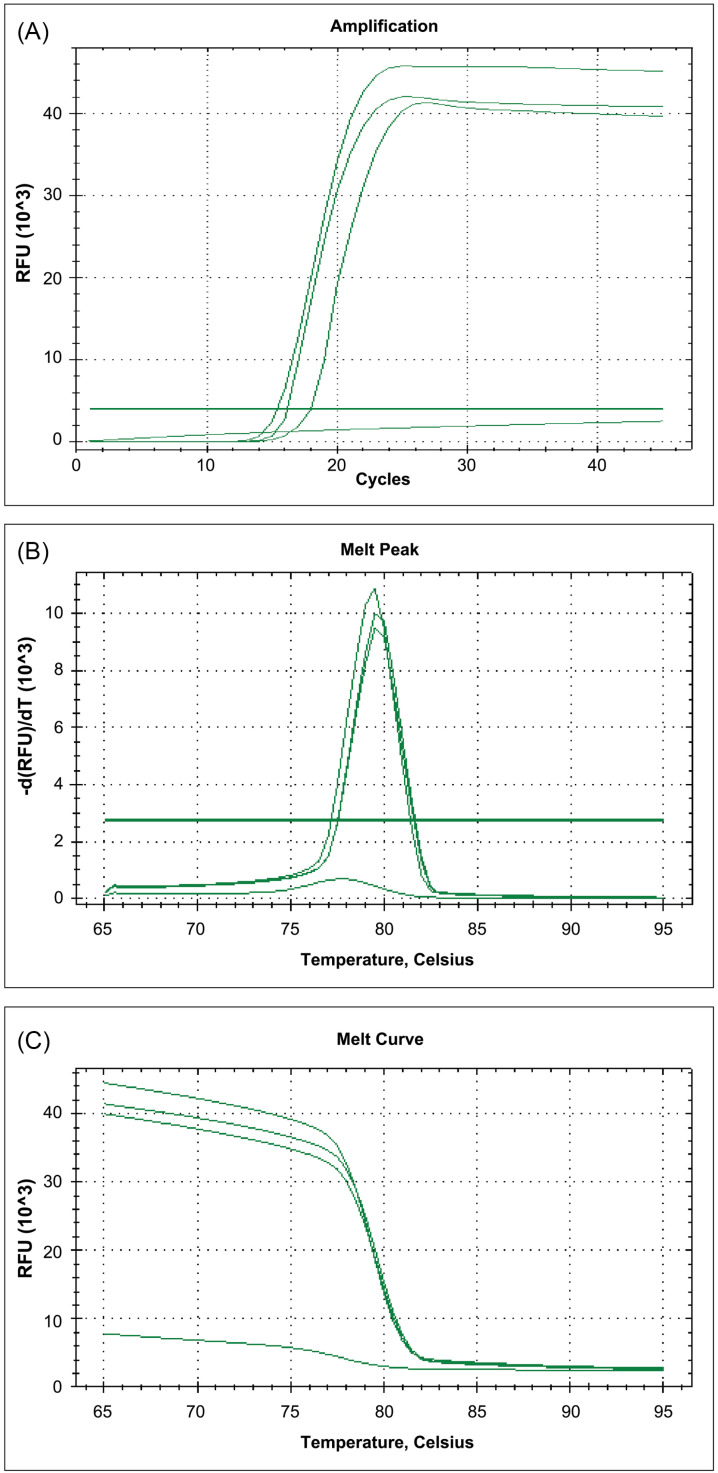
The fluorescence signals in the analysis of the sensitivity of rRT-LAMP. A) The amplification curves in the evaluation of the analytical sensitivity based on the SARS-CoV-2 RNA standard material at the concentrations of 5,203, 4,000, and 3,000 copies/mL. B) The melting temperature of the amplified products in the melting curve analysis showing the amplification specificity targeting SARS-CoV-2 *orf8.* C) The change in fluorescence detected during the melting curve analysis.

The rRT-LAMP assay discussed in this study successfully amplified SARS-CoV-2 RNA at 63 °C within 45 min using the selected rRT-LAMP primer set from our previous study. The kinetic aspect of the amplification reaction could be monitored, and the results could be identified by measuring fluorescent signals in real-time via the increased RFU per cycle in the CFX96^TM^ real-time PCR detection system. Figure 1A and Figure 2A show the examples of amplification results that were displayed on the monitor screen. The reaction threshold set above the baseline in the exponential amplification enabled the determination of positive or negative results. RFU values above 4.0 × 10^3^ on the real-time PCR instrument were considered positive with the amplification curve below 45 cycles. As the reaction progressed, the levels of fluorescence in SARS-CoV-2 positive samples began to increase with incubation time, then the fluorescence peak typically persisted (Figure 1A). There were no amplifications or mis-amplifications in negative controls or negative samples.

### Melting temperatures of the amplicons

After the completion of the rRT-LAMP reaction, the melting curve analysis could be performed in all positive samples, including the positive controls. The amplification specificity could be characterized using the melting curve analysis. The results showed that the rRT-LAMP amplified products provided a single specific melting curve at temperatures ranging from 79.5–81.0 °C ± 1.0 °C (Figure 1B and Figure 2B).

In the melting curve chart, the fluorescence was plotted against the temperature; the change in fluorescence was then plotted against the temperature to obtain a clear view of the melting dynamics in positive samples containing SARS-CoV-2 (Figure 2C). From the fluorescence plot, the level of fluorescence increased during amplification. After post-amplification, heating resulted in a decrease in fluorescence according to the dissociation of the fluorescent dye from the amplified product (Figure 2C). Notably, the primer dimer was not detected according to any other melting curves in the specificity analysis.

### Analytical sensitivity and specificity of the rRT-LAMP

The analytical sensitivity could be determined using a set of dilutions for known concentrations of SARS-CoV-2 RNA prepared using AccuPlex™ SARS-CoV-2 RNA reference materials. This rRT-LAMP was found to be capable of detecting purified SARS-CoV-2 RNA templates in an order of RNA concentrations (Figure 2A). Based on the amplification curves and melting curve analyses, the lowest concentration of the initial SARS-CoV-2 RNA template tested at 3,000 copies/mL was detected, as demonstrated by the detectable amplification curve after the reaction. Therefore, the sensitivity of the rRT-LAMP for SARS-CoV-2 *orf8* amplification was roughly estimated to be as low as 3,000 copies/mL or 15 copies per reaction. Figure 2A depicts the result of the analysis of the analytical sensitivity of the rRT-LAMP.

**Figure 3:**
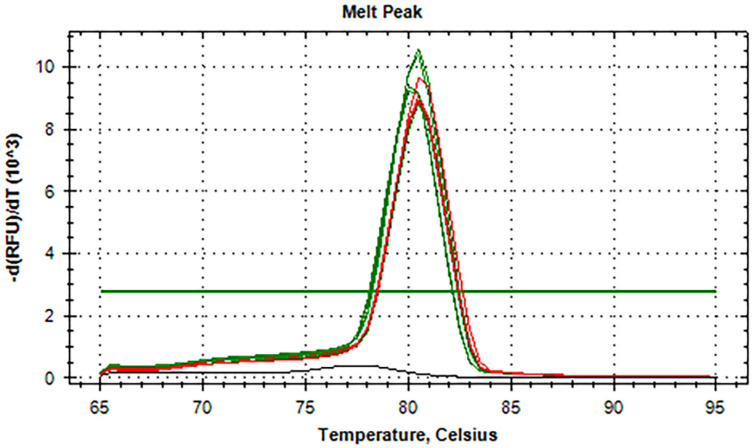
The melting temperatures (Tm) in comparison to the detection of different strains of SARS-CoV-2 and BA.2.75 as a reference known variant. Green colored peaks = Tm of different isolates of BA.2.75 variants; Red colored peaks = Tm of different unknown SARS-CoV-2 strains.

The specificity of primers used in this study was evaluated extensively and addressed elsewhere^21^. In this study, testing results with related viral RNA showed that the rRT-LAMP was specific to SARS-CoV-2. This happened because no amplification curves were detected via fluorescent signals, indicating no amplified products in the reactions containing RNA templates from other related viruses. Furthermore, no amplification curves were observed in the negative control samples. These negatives indicated that the rRT-LAMP assay was dependable for the SARS-CoV-2 RNA template, suggesting the specificity of the test for SARS-CoV-2. The assay also showed that it could detect six isolates of the BA.2.75variant as a reference SARS-CoV-2 genotype with the melting temperature (T_m_) of 80.5 °C, which could be compared to other unknown SARS-CoV-2 strains tested (Figure 3).

### Clinical performance of the rRT-LAMP assay for the detection of SARS-CoV-2

A total of 262 NPS residue samples with corresponding rRT-PCR results were received from the hospital anonymously. NPS residuals comprising 73 rRT-PCR negative samples and 189 rRT-PCR positive samples with varied Ct values were further tested by this new rRT-LAMP assay. A total of 149 samples had Ct values ≤ 30 cycles, and 40 samples had Ct values > 30 cycles, according to the average Ct values (Ct range 9.09–37.97 cycles). The rRT-LAMP results were completely obtained from these tested samples and compared to those of rRT-PCR. Figure 1 shows the representatives of rRT-LAMP results for the detection of SARS-CoV-2 in the tested samples. All rRT-LAMP-positive samples showed the positive amplification curve within 30 min. The minimum time for rRT-LAMP positive detection was recorded at 9.16 min.

When compared with rRT-PCR, the rRT-LAMP results showed that 159 out of 189 (84.13%; 95% CI: 71.56-98.27%) positive samples were positive by rRT-LAMP, and all 73 (100%, 95% CI: 78.38–100%) negative samples were rRT-LAMP negative (Table 1). In comparison to rRT-PCR, the overall diagnostic accuracy, sensitivity, and specificity, including PPV and NPV of the rRT-LAMP were 88.55% (n = 232/262; 95% CI: 77.52-100%), 84.13% (n = 159/189; 95% CI: 71.56-98.27%), 100% (n = 73/73; 95% CI: 78.38-100%), 100% (n = 159/159; 95% CI: 85.06-100%), and 70.87% (n = 73/103; 95% CI: 55.55-89.11%), respectively. Table 1 summarized the rRT-LAMP results and rRT-LAMP assay clinical performance in comparison with rRT-PCR detection.

**Table 1:** The diagnostic performance of the rRT-LAMP in comparison with the rRT-PCR for the detection of SARS-CoV-2.

Tested samples (n = 262)		rRT-PCR		Accuracy (95% CI)	Sensitivity (95% CI)	Specificity (95% CI)	PPV (95% CI)	NPV (95% CI)
		Positive	Negative					
**rRT-LAMP**	**Positive (n = 189)** **Negative (n = 73)**	159 30	0 73	88.55% (n = 232/262) (77.52-100%)	84.13% (n = 159/189) (71.56-98.27%)	100% (n = 73/73) (78.38-100%)	100.0% (n = 159/159) (85.06-100%)	70.87% (n = 73/103) (55.55-89.11%)

n = number.

### Comparative results of rRT-LAMP and rRT-PCR based on Ct values for the detection of SARS-CoV-2

To further assess the clinical performance of the rRT-LAMP assay, the results of rRT-LAMP and rRT-PCR were compared based on the rRT-PCR Ct values targeting the *RdRp, E,* and *N* genes. Comparative results revealed high sensitivity (98.01–100%) in positive samples with rRT-PCR Ct values ≤ 30 cycles (Table 2). Among samples with Ct values > 30–35 cycles, the sensitivities of the rRT-LAMP reduced to 34.29% (*n* = 12/35) for the *RdRp* gene, 26.47% (n = 9/34) for the *E* gene (*n* = 9/33), and 33.33% (*n* = 11/33) for the *N* gene, respectively. The sensitivity reduction was frequently observed as rRT-PCR Ct values increased (Table 2). Meanwhile, there were a small number of positive samples with Ct values > 35 cycles; few samples among these could be detected by the rRT-LAMP, corresponding to a sensitivity reduction of less than 25%. The best sensitivity of the rRT-LAMP was 100% among positive samples with Ct values ≤ 30 cycles targeting the *RdRp* gene. Table 2 shows the amplification results and sensitivities of rRT-LAMP based on the Ct values of the positive rRT-PCR detection.

**Table 2:** The detection results and sensitivities of the rRT-LAMP assay for the detection of SARS-CoV-2 based on rRT-PCR Ct values.

n =189	Ct	rRT-PCR positive				Targets
		n (Sensitivity)				
		≤ 30.00	30.01 - 35.00	35.01-40.00	> 30	
rRT-LAMP	Positive	145/145 (100%)	12/35 (34.29%)	2/9 (28.57%)	14/44 (31.82%)	*RdRp*
	Negative	0	23	7	30	
	Positive	148/151(98.01%)	9/34 (26.47%)	1/4 (25.00%)	10/38 (26.32%)	*E*
	Negative	3	25	3	28	
	Positive	147/149 (98.66%)	11/33 (33.33%)	1/7 (14.29%)	12/40 (30.00%)	*N*
	Negative	2	22	6	28	
	Positive	147/149 (98.66%)	11/36 (30.56%)	1/4 (25.00%)	12/40 (30.00%)	Average Ct
	Negative	2	25	3	28	(3 targets)

## DISCUSSION

This study reports the development and evaluation of a simple rRT-LAMP assay coupled with a melting curve analysis to rapidly detect SARS-CoV-2. Since the rRT-PCR is the gold standard for COVID-19 diagnosis, it was urgently established in Thailand for detecting SARS-CoV-2 during the initial outbreak of COVID-19, with real-time PCR instruments becoming available across the country. In this study, we have further developed the rRT-LAMP as an alternative that should be used with the available real-time thermal cyclers. The advantages of the rRT-LAMP include the real-time monitoring of the amplification reactions and the agility of test results, which are available in less than an hour. Moreover, real-time readouts can prevent subjective interference from affecting results in cases in which it may be difficult to distinguish the color change in samples by the naked-eye visualization of colorimetric RT-LAMP assays. The measurement of fluorescence has been reported to occasionally increase in reaction sensitivity, especially in calling positives that would have been indeterminate by visual detection due to the low intensity of the color change^18^. Besides, real-time reading of rRT-LAMP results using fluorescence measurement could provide quantitative data that were more amenable to high-throughput detection, like rRT-PCR.

A previous study has described the diagnostic value of RT-LAMP and RT-PCR^23^. LAMP is considered a simple nucleic acid amplification via isothermal amplification. This rRT-LAMP is simple since it employed ready-use reagents, which are convenient when assembling the reactions. The assay integrates reverse transcription, LAMP amplification, and real-time fluorescence detection into a one-tube reaction to facilitate the diagnosis of COVID-19 at 63 °C in less than 45 min. The comparative rRT-LAMP results demonstrated good diagnostic performance consistent with the colorimetric RT-LAMP using the same primer set with 87.0% sensitivity and 100% specificity^21^. The primers targeting a specific portion of SARS-CoV-2 *orf8* were chosen because they targeted a conserved, less mutated region in the most recent variants, which was specific to SARS-CoV-2 at the time of sequence analysis^21^. Although strain typing has not been performed in the study, the Delta and Omicron lineages, each with its variants, accounted for the great majority of COVID-19 cases according to the information on circulating SARS-CoV-2 lineages in Thailand at the time of sample collection.

In Mae Sot district, Tak province, the strains of SARS-CoV-2 that were most commonly reported during the time of the study were Omicron variants (BA.1, BA.1.1, BA.2, BA.2.9 subvariants), Delta, and Alpha variants. Considering the highly conserved region in the target sequences, this rRT-LAMP assay was expected to capably detect the SARS-CoV-2 regardless of the SARS-CoV-2 variants. It would be interesting to test different strains of SARS-CoV-2 or known variants in comparison, even though *in silico* analysis can predict the binding effectiveness of the primer to the target sequences. The rRT-LAMP assay could detect different isolates of the SARS-CoV-2 BA.2.75 variant compared to unknown genotypes tested, as shown in Figure 3, based on melting curve analysis. Only samples containing SARS-CoV-2, including a positive control in the test, exhibited a positive reaction indicating the specificity of the test. In general, the LAMP method is highly specific due to its use of 4-6 primers targeting 6-8 target sequences. In this development, the melting curve analysis was applied to verify amplification specificity. It should be noted that all negative samples had no detectable melting curves, and RFU values were less than 4.0 × 10^3^ units in negative reactions. The melting curve analysis also showed that our primers did not amplify non-specifically and did not form dimers, confirming that our method was specific to the SARS-CoV-2 target sequence. The post-amplification melting curve analysis provided a simple method to screen rRT-LAMP reactions for primer-dimer artifacts and to verify reaction specificity. In addition, the characterization of rRT-LAMP products using melting curve analysis could reduce the need for time-consuming gel electrophoresis. It was demonstrated that the specific amplicons resulting from the rRT-LAMP reactions had a typical melting plot, while a corresponding melting temperature was observed in a single peak profile. The method could be applied to detect other viruses or bacteria by employing certain primers targeting unique sequences specific to each pathogen under investigation.

Regarding the reaction time, the waiting time for the results of the rRT-LAMP assay was shorter than the colorimetric RT-LAMP and rRT-PCR, which usually provided results after 1.5 to 3 hours. Generally, LAMP assays are two to three times faster than PCR, which saves equipment and personnel time. We found that this assay could detect SARS-CoV-2 starting from 9–10 min while the reactions were monitored for 45 min. Since all rRT-LAMP positive samples exhibited positive amplification curves within 30 min, the minimum time for the rRT-LAMP reaction could be reduced to at least 30 min to save time. According to the study of Alhamid *et al*.^18^, the colorimetric and fluorometric comparison using the same primer set revealed that the latter was faster than the former. Therefore, another positive feature of rRT-LAMP is its quickness. As there are currently rapid RT-PCR tests with reaction times of about 35 min, the execution time of those tests compared to the rRT-LAMP assay may not be different. In this context, the rRT-LAMP, including other in-house assays with a short reaction time, could be alternatives in places where those rRT-PCR tests are not available. Similarly to the colorimetric RT-LAMP, rRT-LAMP reagents are inexpensive, with comparable costs between the two assays. The estimated cost of our rRT-LAMP would be similar to that reported by Iqbal *et al*.^24^ since the same real-time LAMP reagents are used. In terms of cost analysis, the average cost per-test of rRT-LAMP was reported to be about $8.45 when considering all related expenses, including RNA extraction, LAMP reagents, consumable supplies, and labor cost^24^. Considering the running time, assay cost, and sensitivity, the rRT-LAMP assay could be an alternative laboratory test. It is feasible to set up rRT-LAMP in places where real-time PCR instruments are available. Since naked eye-observation provides a promising on-site point-of-care testing method, including limited-resource laboratories, rRT-LAMP results using the WarmStart reagent could be alternatively read under ultraviolet light^24^. Interestingly, the readings were consistent with the diagnostic sensitivity of 86.0%^24^. If real-time thermal cyclers are unavailable, the reaction can be carried out using a heating block or water bath and read by visualization.

Regarding test sensitivity, the rRT-LAMP could detect SARS-CoV-2 RNA as low as 3,000 copies/mL or 15 copies of viral RNA per reaction, suggesting that this rRT-LAMP assay was sensitive and similar to the colorimetric LAMP we developed earlier^21^. The evaluation showed that the rRT-LAMP had an overall clinical sensitivity of 84.1% compared to the 87.0% sensitivity of our colorimetric RT-LAMP^21^. Considering the recent study by Alhamid *et al.* ^18^, the fluorometric rRT-LAMP targeting one region of the SARS CoV-2 genome had 92.2% sensitivity compared to 89.5% sensitivity exhibited by the colorimetric RT-LAMP assay using the same primer set. The reason for the lower sensitivity detected by the rRT-LAMP compared to the colorimetric RT-LAMP could be the long-term storage of some samples, resulting in the partial degradation of RNA materials in them. However, the sensitivity of this rRT-LAMP was in the acceptable range of 80% sensitivity with 97% specificity, respectively, according to WHO's criteria for alternative COVID-19 molecular tests^25,26^. Therefore, it has the potential for use as an alternative to rRT-PCR. In addition to WHO guidelines for SARS-CoV-2 diagnostic testing, it was recommended that the molecular tests should target at least two independent regions of the viral genome^27^. Like rRT-PCR assays, rRT-LAMP allows for simultaneous multiple target amplification, enabling concurrent detection of two or more target sequences. Multiplex assays may perform better in terms of sensitivity when tested on samples with low viral load or in reducing the risk of false-negative results. However, developing rRT-LAMP assays that target at least two regions or the multiplex rRT-LAMP would be much more complicated^28^. As we aimed to develop a simple rRT-LAMP assay similar to the colorimetric LAMP, the test was designed to target only one highly conserved region of the *orf8*.

Based on rRT-PCR Ct values, the clinical performance of the rRT-LAMP was assessed in further detail. In comparison to rRT-PCR, for samples with Ct values less than 30 cycles, the rRT-LAMP reached 100% sensitivity. Remarkedly, a sensitivity reduction was observed in samples with high Ct values or very low viral loads. The variation in sensitivity based on Ct values indicated that the viral load in each sample strongly affected the sensitivity of the test. Other factors also had an impact on it, such as specimen type and specimen processing^19,20^. The standard reference used in the evaluation studies is another factor^23^. Few studies on the rRT-LAMP assays are available; however, they have been shown to have a high sensitivity for detecting SARS-CoV-2^18,24,29^. A limitation of this rRT-LAMP is the low sensitivity in clinical samples with low viral load or Ct values > 30. This sensitivity might be similar to that found in simple tests, such as rapid antigen tests (RATs). Apparently, the sensitivity of various RATs appears to be highly variable. A high sensitivity of the RAT compared to the RT-PCR was reported^30^; despite their ease of use and high sensitivity, many RATs might not be generally available due to the decline of COVID-19 cases. In this regard, the in-house assays may serve as alternatives available for local testing. Overall, the clinical sensitivity of this rRT-LAMP was within the acceptable range, and the procedure was simple enough to perform.

The limitations of this study are as follows: firstly, NPS residual samples were stored, therefore, some portions of RNA material might have degraded and affected the sensitivity of the test; secondly, a limited number of the known variants of SARS-CoV-2 were tested to compare the detection of different strains. In addition, we could not assess the potential of the rRT-LAMP assay to detect SARS-CoV-2 in a variety of specimens. However, the strength of the study was that a large number of positive samples with varying Ct values were available for evaluation. Despite the limitations mentioned, the study demonstrated the performance and the advantages of the rRT-LAMP, which has potential as an alternative for detecting real-time amplification of SARS-CoV-2 RNA.

## CONCLUSION

The developed rRT-LAMP showed a good performance for the rapid detection of SARS-CoV-2 with an overall sensitivity of 84.13% and 100% specificity. The highest sensitivity was about 98–100% in samples with Ct values ≤ 30 cycles, while the melting curve analysis provided a function to confirm amplification specificity. The study highlighted the advantage of real-time amplification detection of the rRT-LAMP, which was similar to rRT-PCR but with less technical requirements, shorter duration, and lower cost. Its rapidity, sensitivity, high specificity, low cost and ease of real-time amplification detection make the rRT-LAMP assay a promising alternative molecular test. The assay can expand the range of laboratory-based molecular tests available for rapid SARS-CoV-2 detection.
